# Post-treatment haemolysis in African children with hyperparasitaemic *falciparum* malaria; a randomized comparison of artesunate and quinine

**DOI:** 10.1186/s12879-017-2678-0

**Published:** 2017-08-17

**Authors:** C. Fanello, M. Onyamboko, S. J. Lee, C. Woodrow, S. Setaphan, K. Chotivanich, P. Buffet, S. Jauréguiberry, K. Rockett, K. Stepniewska, N. P. J. Day, N. J. White, A. M. Dondorp

**Affiliations:** 1 Mahidol-Oxford Tropical Medicine Research Unit, Faculty of Tropical Medicine, Mahidol University, Bangkok, Thailand; 2 Centre for Tropical Medicine and Global Health, Nuffield Department of Medicine, University of Oxford, Oxford, UK; 3 Kinshasa School of Public Health, University of Kinshasa, Kinshasa, Democratic Republic of the Congo; 4 Department of Clinical Tropical Medicine, Faculty of Tropical Medicine, Mahidol University, Bangkok, Thailand; 5 Institut National de la Transfusion Sanguine, Université Paris Descartes/INSERM UMR_S 1134, Paris, France; 6 Laboratoire d’Excellence GR-Ex, Paris, France; 7 Assistance Publique–Hôpitaux de Paris, Centre National de Référence du Paludisme, Paris, France; 8 Wellcome Trust Centre for Human Genetics, University of Oxford, Oxford, UK; 9 WorldWide Antimalarial Resistance Network, Oxford, UK

## Abstract

**Background:**

Parenteral artesunate is the treatment of choice for severe malaria. Recently, haemolytic anaemia occurring 1 to 3 weeks after artesunate treatment of *falciparum* malaria has been reported in returning travellers in temperate countries.

**Methods:**

To assess these potential safety concerns in African children, in whom most deaths from malaria occur, an open-labelled, randomized controlled trial was conducted in Kinshasa, Democratic Republic of Congo. 217 children aged between 6 months and 14 years with acute uncomplicated *falciparum* malaria and parasite densities over 100,000/μL were randomly allocated to intravenous artesunate or quinine, hospitalized for 3 days and then followed for 42 days.

**Results:**

The immediate reduction in haemoglobin was less with artesunate than with quinine: median (IQR) fall at 72 h 1.4 g/dL (0.90–1.95) vs. 1.7 g/dL (1.10–2.40) (*p* = 0.009). This was explained by greater pitting then recirculation of once infected erythrocytes. Only 5% of patients (in both groups) had a ≥ 10% reduction in haemoglobin after day 7 (*p* = 0.1). One artesunate treated patient with suspected concomitant sepsis had a protracted clinical course and required a blood transfusion on day 14.

**Conclusions:**

Clinically significant delayed haemolysis following parenteral artesunate is uncommon in African children hospitalised with acute *falciparum* malaria and high parasitaemias.

**Trial registration:**

ClinicalTrials.gov; Identifier: NCT02092766 (18/03/2014)

**Electronic supplementary material:**

The online version of this article (doi:10.1186/s12879-017-2678-0) contains supplementary material, which is available to authorized users.

## Background

Parenteral treatment with artesunate has been shown to reduce mortality from severe malaria by 35% in adults and 23% in children compared to quinine [1, 2] and is the recommended first-line treatment for severe malaria [3]. Artesunate is well tolerated, safe and has fewer adverse effects than quinine. Until recently the only serious adverse event observed has been a Type-1 hypersensitivity reaction in approximately one per 3000 treatments [4–6]. However, there are now several reports, mainly from temperate countries, of late haemolytic anaemia occurring 1 to 3 weeks after parenteral artesunate treatment of hyperparasitaemic *falciparum* malaria in returned travellers. This has raised general concerns about the safety of these life-saving compounds. Most cases followed intravenous artesunate, although some were reported after intramuscular artemether, intrarectal artesunate and oral artemisinin derivatives [7, 8]. There were no deaths, but blood transfusion was often required. A recent review of published data, from non-immune and semi-immune patients, estimated the incidence of late haemolysis after intravenous artesunate to be 13% (95% CI 9–18%) and the requirement for a blood transfusion at 9% (95% CI 6–14%) [7], although most data are from case reports and case definitions have varied substantially. But returned travellers constitute only a very small fraction of the global burden of severe malaria. Are these observations in travellers of clinical relevance to African children, who carry more than 90% of the global malaria disease burden, and for whom life-threatening anaemia is a common clinical presentation of *falciparum* malaria? Severe malarial anaemia in childhood is associated with significant mortality both in hospital and after discharge [9]. As haemolytic anaemia is the pathological hallmark of all malaria infections, and severe anaemia is a common feature of severe malaria, dissecting disease from drug-associated mechanisms is difficult. To investigate the pathogenesis of anaemia in African children and whether post-artesunate haemolytic anaemia is a significant problem, a randomised comparison was conducted of the immediate and delayed haematological responses to parenteral artesunate and quinine in children hospitalised with high *falciparum* parasitaemias.

## Methods

The trial was conducted at the Kinshasa Mahidol Oxford Research Unit, Kinshasa, the Democratic Republic of Congo. Malaria in this area is endemic and perennial, with minor seasonal variations.

Children with acute *falciparum* malaria were eligible for enrolment if they were aged between 6 months and 14 years old, had body weight > 5 kg, haemoglobin ≥5.0 g/dL and parasitaemia ≥100,000 *falciparum* parasites/μL. Patients with any sign of severe malaria [3] (although hyperparasitaemia alone was not an exclusion criterion [10]), a history of hypersensitivity or contraindication to the use of artesunate or quinine, a clear history of adequate antimalarial treatment within 24 h, or the presence of a significant other illness, were excluded. The study was explained to caregivers in French or Lingala and they were requested to sign a consent form to allow their child’s participation in the study.

The study was an open-labelled randomised comparison of the hematological effects of parenteral antimalarial treatment with either artesunate or quinine. Computer randomisation, in blocks of 20, was prepared by a study statistician. Treatment allocation was concealed in sequential opaque envelopes prepared by an independent person. The intervention was assigned by the study nurse, after the doctor confirmed eligibility and the caregivers had signed the informed consent. After a full clinical examination, patients were randomised to receive intravenous (IV) artesunate (AS) 2.4 mg/kg body weight bolus (Guilin Pharmaceutical, Guangxi, China) immediately and at 12, 24, 48 and 72 h or quinine dihydrochloride (QN) 20 mg salt/kg body weight (Sterop, Brussels, Belgium) infused over 4 h, followed by 10 mg/kg body weight every 8 h infused over 2 h until 72 h.

Parenteral treatment was followed by a full course of oral artemether-lumefantrine (Coartem®, Novartis); the first dose was directly observed and the remaining doses were given with instructions to the caregiver for administration at home. Patients were followed-up weekly for 6 weeks. Any recurrent episodes of malaria were treated with fixed-dose amodiaquine-artesunate tablets (Winthrop®, Sanofi). Blood transfusion was given when haemoglobin was ≤5 g/dL regardless of clinical symptoms, or if the haemoglobin was ≤7 g/dL and there were one or more clinical features of severity present [3].

Malaria blood films were prepared at admission, 0 (baseline), 6, 12 h, then every 12 h until 2 consecutive microscopy negative blood films and at each follow-up visit. Parasites were counted using standard methods. Hemoglobin (Hb) and haematocrit (Hct) were assessed at the same time-points as the blood films using HemoCue Hb201+ (Angelholm, Sweden) and Hawksley Haematospin 1400 (Hawksley & Sons, Ltd. UK). Reticulocytes were counted as a percentage of total RBCs at 0, 72 h and weekly.

Ring-infected Erythrocyte Surface Antigen (RESA)-positive, *falciparum*-negative RBCs (*once infected* or *oi*-RBCs) were counted *per* 1000 RBCs at 0, 24, 48, 72 h and then weekly by immunofluorescence microscopy [11]. Total and differential white blood cell (WBC) counts were measured at 0, 72 h and weekly by QBC Star™.

Hemoglobin HbS [rs334] and A^−^ glucose-6-phosphate dehydrogenase deficiency defined by G6PD 376 (rs1050829) and G6PD 202 (rs1050828) were characterised from genomic DNA at the Wellcome Trust Centre for Human Genetics using Agena MassArray IPLEX platform and multiplexed PCR assay [12].

Quantification of plasma *Pf*HRP2 by ELISA (Cell-labs, Australia) and plasma lactate dehydrogenase (LDH; Olympus AU400 Automated chemistry analyser) was performed in Bangkok, Thailand. Laboratory technicians were blinded to study treatments.

The prespecified primary endpoint was the proportion of patients who developed late anaemia, defined as a reduction of ≥10% in haemoglobin compared with any previous measurement between days 7 and 42 after starting parenteral antimalarial treatment. Secondary endpoints included: the proportions of patients requiring blood transfusion, the fractional decreases in haemoglobin in the first week of treatment, plasma LDH as a measure of haemolysis, reticulocyte and pitted erythrocyte counts, parasite clearance times, and frequency of serious adverse events.

The proportion of intravenous artesunate treated patients with delayed anaemia was assumed to be 20% [13]. To provide 90% power and 95% confidence to detect a difference of 20% vs. 4%, with 10% loss to follow-up, 108 children per arm were recruited.

Comparisons between groups used the Student’s t-test or Mann-Whitney U test, depending on distributions. Kendall’s Tau (τ) or Pearson’s correlation coefficient (rho_p_) assessed associations between continuous variables. For more than two groups, the Kruskal-Wallis test was used. AUCs were estimated using the trapezoid rule. Hemoglobin, *oi*-RBC and parasite count values were censored on the day of malaria recrudescence or blood transfusion.

Parasite clearance was assessed as the time for the parasite count to fall by 50% (PC_50_), the parasite clearance half-life (PC_1/2_) [14] and the interval until patients became parasite negative by microscopy (PCT).

Clearance of *oi*-RBC was estimated by modelling the decline in log-transformed *oi*-RBC over time in a random-effects model, with variable intercept and slope. Mono- and biexponential decay curves were considered. The final model was selected based on the Akaike Information Criterion (AIC). Levels of *oi*-RBC below the detection limit were excluded from the analysis.

To quantify associations with significant predictors of haemoglobin concentration, linear regression was used with random effects fitted for both the intercepts and slopes of Hb, [log] *oi*-RBC, LDH and [log] reticulocytes. Fixed covariates included sex, age, homozygous/hemizygous G6PD deficiency, sickle cell disease trait, [log] parasitaemia on admission, splenomegaly on admission, malnutrition (a composite of underweight, wasted and stunted) and study drug treatment. Two models were considered: the first to evaluate associations with haemoglobin changes from day 0 to 7 (which included Hb at hour 0 as a covariate), the second to evaluate associations from day 7 to 28.

Safety reporting was performed according to the ICH Harmonized Tripartite Guideline for GCP (1996). The study was approved by the Oxford Tropical Medicine Ethics Committee and the University of Kinshasa, School of Public Health institutional review board. Data were entered using MACRO InferMed and analysed using STATA v14.0. (ClinicalTrials.gov; Identifier: NCT02092766).

## Results

**Fig. 1:**
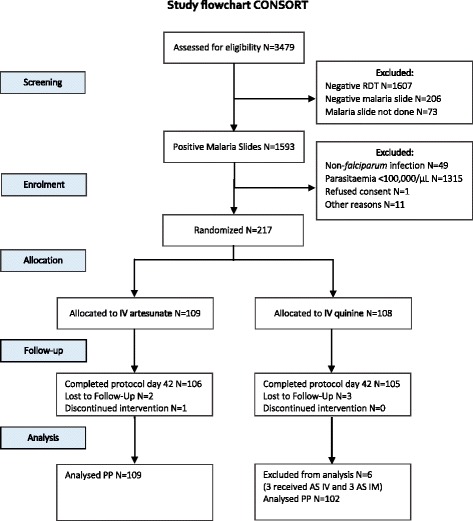
Study flowchart

Between June 2014 and March 2015, 217 children were enrolled (Fig. 1). One study patient, whose parents withdrew their consent the first day of hospitalization, was replaced (AS). Four patients developed severe malaria in the first 24 h (AS 1; QN 3); for those allocated to QN, the treatment was changed to IV AS. In three other cases (QN), difficulties in obtaining venous access prompted a change to intramuscular (IM) artesunate. Thus, hematological outcomes were analysed in 211 children (AS 109; QN 102). The two groups had similar demographic, clinical and laboratory characteristics on admission (Table 1). Five patients were lost to follow-up after day 7 (AS 2; QN 3), all remaining patients were followed until day 42. Most patients (75.6%) had an adequate clinical and parasitological response at day 42; forty-one (18.9%) had a recurrent episode of malaria with a similar rate in the two arms (log-rank test *p* = 0.80) (Additional file 1: Table S1).

**Table 1:** Demographic, clinical and parasitological data at admission by treatment arm (as per allocation)

	AS	QN
Number of admitted patients	109	108
Female: Male	59:50	52:56
Mean (range) age (months)	70.3 (9–167)	66.8 (6–153)
Mean (SD) weight (kg)	18.6 (7.8)	18.0 (6.8)
Mean (SD) height (cm)	109.5 (21.8)	107.7 (20.6)
Malnourished (%)	31.2	23.5
A^−^ G6PD allele frequency (%)	18.3	15.7
Sickle cell trait allele frequency (%)	6.9	6.9
Palpable splenomegaly (%)	49.5	40.7
Hepatomegaly (%)	28.4	22.2
Median (range) Axillary temperature (°C)	37.1 °C (36.0–40.4)	37.0 °C (36.0–39.9)
Mean (SD) Systolic blood pressure (mmHg)	89 ± 8	89 ± 9
Mean (SD) Diastolic blood pressure (mmHg)	54 ± 8	53 ± 8
Mean (SD) Heart Rate (beats/ min)	125 ± 21	123 ± 21
Mean (SD) Respiratory Rate (breaths/min)	36 ± 8	34 ± 6
Mean (SD) WBC count, 10^3^/ΜL	8.0 (3.5)	7.3 (3.1)
Mean (SD) haemoglobin, g/dL	10.4 (1.7)	10.3 (1.8)
Mean (SD) haematocrit (%)	31.3 (4.9)	30.7 (5.3)
Median P. falciparum parasitaemia/μL (range)	179,608 (100,480–1,386,624)	177,473 (101,108–840,264)
Geometric mean P. falciparum density (/μL) (95% CI)	201,450 (181,764–223,268)	199,070 (181,496–218,346)
No. patients with >250,000/ΜL (%)	35.8%	41.7%
Geometric mean plasma PfHRP2 ng/mL (95% CI)	558.8 (*N* = 51) (369.7–844.6)	564.7 (*N* = 47) (344.7–925.1)
Median oi-RBC count/μL (range)	10,927 (0–104,499)	11,680 (0–145,947)

### Dynamics of infected and once-infected erythrocytes

Admission geometric mean parasitaemias were similar in the two treatment groups (Table 1; *p* = 0.89) and correlated inversely with age (rho_p_ = −0.186, *p* = 0.007). Parasites cleared significantly faster in patients treated with AS compared to QN: PC_50_ median (range) 5.1 h (0.08–15) vs. QN 15 h (0.04–28.6), *p* = 0.0001; PC_1/2_ median (range) AS 1.8 h (1.0–3.2) vs. QN 2.8 h (1.0–9.7), *p* = 0.0001. The median (IQR) PCT was 2 days in the AS arm (1–2) vs. 3 days in the QN arm (2–3), *p* < 0.001.

**Fig. 2:**
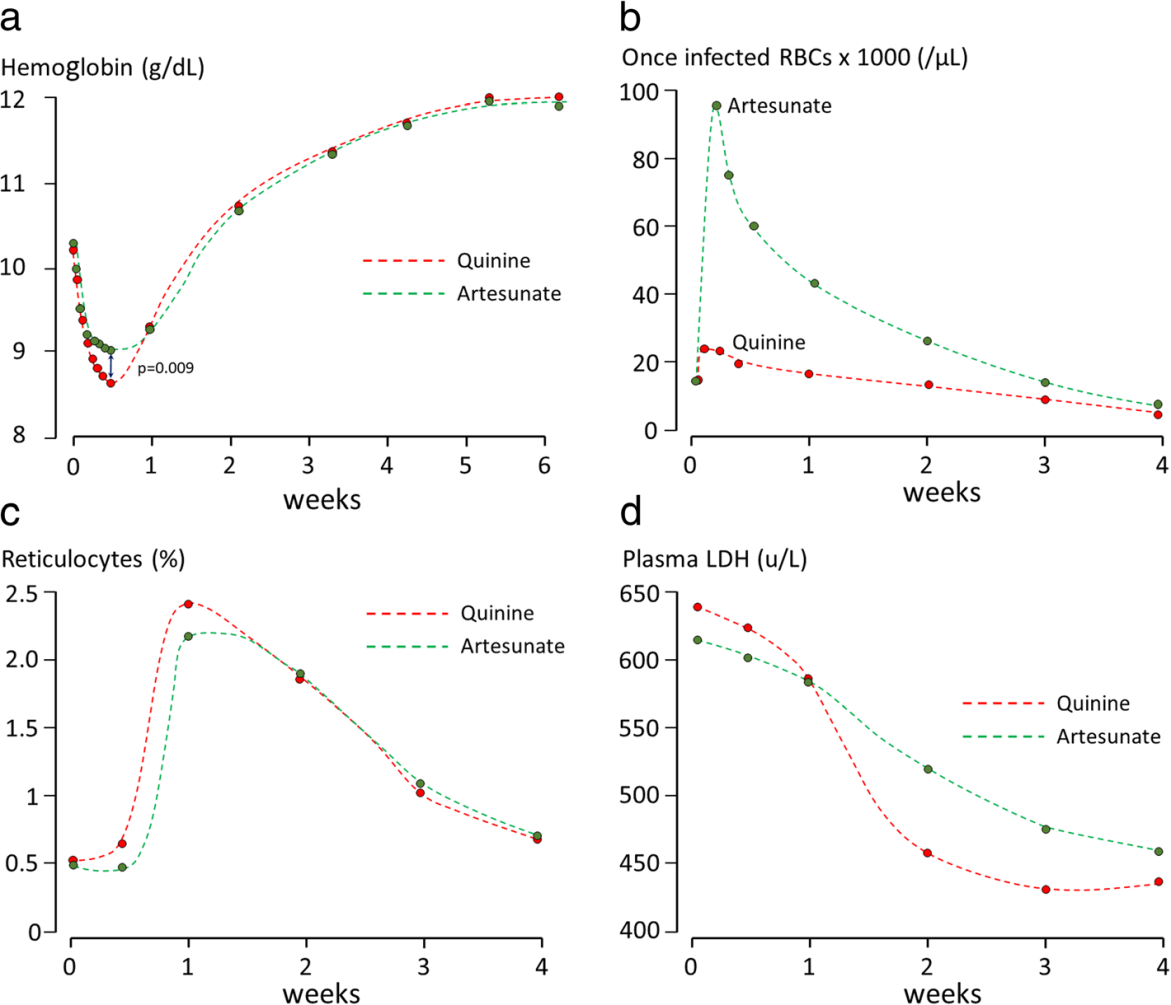
Mean haemoglobin **a**, median *oi-*RBCs **b**, reticulocyte counts **c** and LDH **d**, by treatment, over time

The *oi*-RBC counts peaked at 24 h in the AS group and at 48 h in the QN group (IQR 1–2 days for both arms; *p* = 0.09; Fig. 2b). The median post-treatment *oi*-RBC peak was over three times higher with AS compared to QN: 100,417/μL vs. 31,714/μL (*p* < 0.001, Additional file 1: Table S2). The ratio between maximum number of *oi*-RBC and baseline parasitaemia was 59% with AS vs. 14% with QN (*p* < 0.001).

A first-order biexponential decay best described the decline in *oi*-RBC values: the initial decline was similar between treatments with a half-life of 2.0 (95% CI 1.7–2.3) days. The terminal elimination phase began around 2 days after peak values with a half-life which was shorter following AS than QN: 10.0 (95% CI 9.0–11.1) days vs.14.8 (95% CI 12.6–18.0) days (*p* < 0.001).

### Anaemia

On admission 74% of children were anaemic according to WHO cut-offs by age-group [15]: 23% mild, 47% moderate and 4% severe. The mean haemoglobin concentration was similar in the two arms (Additional file 1: Table S3 & Fig. 2a, p=0.600) and correlated positively with age (rho_p_ = 0.36, *p*<0.001). Malnourished children had a significantly lower mean (SD) Hb 9.7 g/dL (1.7) vs. 10.4 g/dL (1.6); *p* = 0.006. Children homozygous/hemizygous for G6PD deficiency (*N*=22) also had a lower mean (SD) values, 9.7 g/dL (1.4) vs. 10.3 g/dL (1.7), although the difference was not significant (*p*=0.12).

Four patients received blood transfusions, two in the first 12 h (QN), one at day 5 (AS) and one at day 14 (AS). The single case given a late blood transfusion was a 6-year-old boy admitted with a parasitaemia of 145,068/μL which increased abruptly to 887,992/μL in the 25 min interval to the start of treatment (suggesting highly synchronous schizogony). The following day the patient deteriorated, was unable to sit unaided and became deeply jaundiced; antibiotics were started for suspected bacterial sepsis. During two-weeks’ hospitalisation, his condition slowly improved, but fever persisted and the antibiotics were changed to ceftriaxone. His haemoglobin declined steadily from 11.0 g/dL on admission to 4.7 g/dL on day 14, with a reticulocyte count of 9.6% and plasma LDH of 2126 U/L. The patient was given a blood transfusion and discharged. He was clinically well when reviewed a week later.

The initial reduction in haemoglobin was greater in the QN group: median (IQR) 1.5 g/dL (0.90–2.20) vs. 1.3 g/dL (0.80–1.90) by 48 h (*p* = 0.036), and 1.7 g/dL (1.10–2.40) vs. 1.4 g/dL (0.90–1.95) by 72 h (*p* = 0.009). However, by day 7 the haemoglobin values were similar in the two treatment arms (Fig. 2a).

The Hb nadir was observed before day 7 in 88% of patients (*N* = 185, lowest recorded Hb 4.5 g/dL) and at day 7 in 10% (*N* = 21, lowest recorded Hb 7.3 g/dL). The median (IQR) interval to Hb nadir was 48 h (36–72) with AS and 60 h (48–72) with QN, *p* = 0.192. The mean (SD) Hb nadir values before day 7 were similar, AS = 8.3 (1.4) g/dL vs. QN = 8.1 (1.6) g/dL, *p* = 0.395, as were the nadirs observed at day 7, AS = 9.2 (1.1) g/dL vs. QN = 9.4 (1.2) g/dL, *p* = 0.736.

Reticulocyte counts (haematocrit corrected) increased until day 7 and then decreased gradually in both arms (Fig. 2c). The counts were significantly higher on day 3 in children who received QN, *p* < 0.001. There were no significant differences after day 3.

The primary endpoint, defined a-priori as a reduction of ≥10% in the Hb level at any time between days 7 and 42, was observed in 19/207 patients: 10 children treated with AS, whose median Hb fractional reduction was 13.7% (IQR 12.7–20.7; lowest Hb 4.7 g/dL) and 9 treated with QN, whose median Hb fractional reduction was 12.8% (IQR 11.1–13.4; lowest Hb 9.4 g/dL, *p* = 0.103; Additional file 2: Figure S1 and Additional file 3: Figure S2).

In the cohort of patients who completed the 42-days follow-up, the Hb concentration increased steadily and rose above the admission level in 92.7% of cases (AS 94/105 vs. QN 96/100; *p* = 0.08). In 15 patients (7.3%) the Hb remained below the admission value throughout the follow-up (AS 11; QN 4) and in 4 patients (2%) the nadir occurred after day 7 (AS 3; QN 1; lowest Hb 4.7 and 10.4 g/dL, respectively).

### Measures of haemolysis

Between day 0 and 7 the median plasma LDH concentrations (U*days/L) decreased by similar amounts in the two groups (Fig. 2d): AUCs AS 4224 vs. QN 4380.5 (*p* = 0.73). The median (range) maximum LDH concentration was 688 (328–2215) U/L with AS and 703 (375–1664) U/L with QN.

From day 7 onward the median LDH concentration decreased more in patients who received QN (*p* < 0.05, Fig. 2d). The median (IQR) fractional reduction from day 7 to 14 was 19.4% in the QN arm (6.5–27.7) and 11.8% in the AS arm (0.2–20.9); *p* = 0.005; and between day 14 and 21, 24.1% in the QN arm (12.5–35.3) and 17.9% in the AS arm (6.4–32.4); *p* = 0.12. The median LDH AUC was slightly smaller with QN compared to AS (9863 vs. 10,637; *p* = 0.04) and was positively correlated with the maximum *oi*-RBC count with AS (*p* = 0.045), but not QN (*p* = 0.263).

Some patients had transient fluctuations in the plasma LDH level during the follow-up period. A significant increase (above twice the paediatric reference range ULN [16]) was observed in 21% (23/109) of AS and 12.7% of QN treated patients (13/102, *p* = 0.107).

Children who received AS and later had a significantly elevated plasma LDH concentration were younger (*p* = 0.045), had higher admission parasitaemias (*p* = 0.0042), higher maximum *oi*-RBC counts (*p* = 0.0150), higher reticulocyte counts (*p* = 0.0102) and lower Hb at days 14 and 21 (*p* = 0.0004 and *p* = 0.0139, respectively). In the QN arm there were no differences in these parameters between patients with and without significant LDH elevations, although *oi*-RBC counts were higher in those with normal LDH (*p* = 0.0132).

### Factors affecting anaemia

Factors affecting the change in haemoglobin were assessed by mixed effects modelling. Compared with the remaining patients, children homozygous/hemizygous for G6PD deficiency had an average 0.687 g/dL (95% CI 0.145–1.229) lower Hb values from day 0 to 7, those with palpable splenomegaly had 0.431 g/dL (95% CI 0.113–0.749) lower Hb and malnourished children had 0.379 g/dL lower Hb levels (95% CI 0.024–0.734). Older children had higher Hb levels, 0.111 g/dL (95% CI 0.055–0.161) per year of age. The effect of treatment was borderline: Hb levels in children treated with AS were, on average, 0.312 g/dL (95% CI 0.002–0.627) higher from day 0 to 7, but thereafter the effect of treatment was no longer significant (*p* = 0.087) and only LDH, [log] *oi*-RBC and [log] reticulocytes were associated significantly with haemoglobin changes (Additional file 1: Tables S4-S5).

## Discussion

Anaemia is a consistent feature of *falciparum* malaria and results from the obligatory lysis of infected erythrocytes at schizogony, increased splenic clearance of both infected and uninfected erythrocytes and dyserytropoiesis. The relative contributions of these factors vary with age, immunity and disease severity. In tropical areas malaria anaemia may be compounded by haematinic deficiencies, hypersplenism, and genetic red cell disorders. As severe malarial anaemia can be fatal, it is important to identify preventable contributors. In this study of African children with acute *falciparum* malaria and high parasite densities living in a high transmission area post-artesunate haemolytic anaemia was substantially less common than reported in non-immune returned travellers.

Post-artesunate haemolytic anaemia has been attributed to the increased pitting of drug-damaged malaria parasites from infected erythrocytes [17]. Pitting is the physiological process whereby the spleen removes intraerythrocytic particulate matter, in this case the pyknotic ring-stage intra-erythrocytic parasites, and then returns the resealed red blood cells into the systemic circulation [11]. The life-saving benefit of artesunate over quinine derives from its rapid parasiticidal activity against the circulating ring-stage parasites. This results in a higher rate of parasite clearance and a correspondingly greater production of *oi*-RBC (Fig. 2b). These once-infected red blood cells (*oi*-RBC) have a much shorter survival time (7–14 days) compared with normal erythrocytes in healthy subjects (120 days) or in patients following severe malaria (44 days) [18, 19]. The shorter survival of *oi*-RBC following artesunate probably reflects drug killing and then pitting of developed ring forms whereas background (and quinine associated) pitting may only occur for very young ring stages shortly after merozoite invasion with correspondingly less damage to the erythrocytes. The rapid and synchronous elimination of these *oi*-RBC from the circulation 1 to 2 weeks after the start of antimalarial treatment might result in haemolytic anaemia [17].

In this study, a similar proportion of artesunate and quinine treated patients had a ≥ 10% reduction in haemoglobin, but the degree of anaemia was moderate, although there are small but significant differences in the time course. Patients receiving artesunate had slightly lower initial reductions in haemoglobin and delayed reticulocyte responses. Previous studies also indicated a clinically insignificant temporary suppression of reticulocytosis by artemisinins in the treatment of malaria [20, 21]. More than three times as many ring stage parasites were pitted after treatment with artesunate compared to quinine, with over half returning to the circulation, largely explaining the initially smaller reduction in haemoglobin in artesunate recipients. The ratio of *oi*-RBC to the initial parasitaemia (59%) was comparable to that observed in Malian children (60%) following artesunate, but lower than in returned travellers (78%) [17]. Moreover, in artesunate-treated travelers around 60% of originally parasitized cells were still present in the circulation 7 days after treatment, while in this study only approximately one quarter of the *oi*-RBCs were left at this stage, thereby reducing the number of erythrocytes contributing to late haemolysis. These findings suggest that other, presumably immune mediated, mechanisms leading to infected cell removal contribute to splenic clearance of parasitaemia in African children. The erythropoietic response to malarial anaemia was rapid (peak 7 days) [22], and took place earlier than in patients with less background immunity (14–21 days [17, 19, 23]). Hence in these semi-immune African children the lower contribution of pitting to parasite clearance after artesunate and the earlier reticulocyte response mitigated the loss of haemoglobin associated with the late lysis of *oi-*RBC, whilst more rapid clearance of pitted cells reduced haemolysis occurring after 7 days. Only one artesunate treated patient had haemolytic anaemia severe enough to warrant blood transfusion. This patient had a complicated disease course with suspected concomitant bacterial sepsis treated. Whether the haemolysis in this case was explained only by malaria and artesunate, or whether the presumed sepsis contributed cannot be determined.

Severe delayed haemolytic anaemia was observed in less than 1% of Congolese children with acute *falciparum* malaria and high parasitaemias, which is similar to the frequency reported in West African children with severe malaria [22]. A limitation of this study is that the patients were not clinically severe whereas post-artesunate delayed haemolysis has been reported particularly after severe malaria. Children with severe malaria could not be randomised because artesunate is clearly the better treatment. Nevertheless, the patients studied were at the severe end of the clinical spectrum of hospitalised uncomplicated malaria. Plasma *Pf*HRP2 concentrations on admission, an indicator of parasite burden, were between the values reported for African children with uncomplicated and severe malaria (approximately 150 ng/ml and 1500 ng/ml respectively [24]).

## Conclusions

In conclusion, severe delayed haemolytic anaemia was rare in this population of African children with high parasitaemias. This reassuring evidence contrasts with recent reports form returned travellers and it strongly supports the wider deployment of life-saving parenteral artesunate for the treatment of severe *falciparum* in African children.

## Additional files

Additional file 1: Table S1. Efficacy outcome by treatment arm PCR uncorrected, Per Protocol. **Table S2.** Median and range *oi*-RBCs/μL of blood by day and treatment arm. **Table S3.** Mean, SD and range of haemoglobin 0–42 days by treatment arm. **Table S4.** Linear regression using Hb (g/dL) as outcome and patient as random effect for days 0–7. **Table S5.** Linear regression using Hb (g/dL) as outcome and patient as random effect for days 7–28. (DOCX 24 kb)

Additional file 2: Figure S1. Individual haemoglobin profiles of patients in the QN group with ≥10% reduction after day 7, *n* = 9 (TIFF 24 kb)

Additional file 3: Figure S2. Individual haemoglobin profiles of patients in the AS group with ≥10% reduction after day 7, *n* =10 (TIFF 64 kb)

## Abbreviations

RESA: Ring-infected erythrocyte surface antigen
95% CI: 95% Confidence interval
AIC: Akaike information criterion
AS: Artesunate
AUC: Area under the curve
DRC: Democratic Republic of Congo
G6PD: Glucose-6-phosphate dehydrogenase
Hb: Hemoglobin
Hct: Haematocrit
IM: Intramuscular
IQR: Interquartile range
IV: Intravenous
LDH: Plasma lactate dehydrogenase
*once infected* or *oi*-RBCs: *RESA-positive, falciparum*-negative RBCs
PC_1/2_: Parasite clearance half-life
PC_50_: Time for parasite count to fall by 50%
PCT: Parasite clearance time (by microscopy)
*Pf*HRP2: *Plasmodium falciparum* Histadine Rich Protein 2
PP: Per protocol
QN: Quinine
RBCs: Red blood cells
SD: Standard deviation
WBCs: White blood cells
