# Supplementary material for: Post-treatment haemolysis in African children with hyperparasitaemic falciparum malaria; a randomized comparison of artesunate and quinine
Source: BMC Infect Dis. 2017 Aug 17;17:575. doi: 10.1186/s12879-017-2678-0 (PMC5561573; doi:10.1186/s12879-017-2678-0)
Supplement: Supplementary file 1 — Efficacy outcome by treatment arm PCR uncorrected, Per Protocol. Table S2. Median and range oi-RBCs/μL of blood by day and treatment arm. Table S3. Mean, SD and range of haemoglobin 0–42 days by treatment arm. Table S4. Linear regression using Hb (g/dL) as outcome and patient as random effect for days 0–7. Table S5. Linear regression using Hb (g/dL) as outcome and patient as random effect for days 7–28. (DOCX 24 kb) [file 12879_2017_2678_MOESM1_ESM.docx]

**Supplementary materials (Tables)**

**Table S1. Efficacy outcome by treatment arm PCR uncorrected, Per Protocol**

| Outcome by day 42 | aS | QN | Total |
| --- | --- | --- | --- |
|  | N (%) | N (%) |  |
| Excluded from Per Protocol Analysis* | 0 | 3 (2.8) | 3 |
| Adequate Clinical and Parasitological Response | 85 (78.0) | 79 (73.2) | 164 |
| Early Treatment Failure | 1 (0.9) | 3 (2.8) | 4 |
| Late Clinical Failure | 5 (4.6) | 2 (1.9) | 7 |
| Late Parasitological Failure | 15 (13.8) | 19 (17.6) | 34 |
| Lost to follow-up/withdrawals | 3 (2.8) | 2 (1.9) | 5 |
| Total | 109 | 108 | 217 |

*treated with IM artesunate

**Table S2. Median and range *oi*-RBCs/µL of blood by day and treatment arm**

|  | AS | | | QN | | |
| --- | --- | --- | --- | --- | --- | --- |
| Day | **N** | **Median** | **Range** | **N** | **Median** | **Range** |
| 0 | 108 | 10,927 | 0-104,499 | 101 | 11,681 | 0-145,947 |
| 1 | 106 | 94,577 | 8,290-904,320 | 100 | 24,994 | 0-228,341 |
| 2 | 107 | 73,853 | 0-889,750 | 102 | 24,366 | 3391-197,820 |
| 3 | 107 | 61,042 | 0-796,932 | 98 | 17,898 | 0-230,790 |
| 7 | 104 | 44,714 | 0-786,758 | 100 | 14,318 | 0-150,218 |
| 14 | 105 | 21,854 | 0-367,882 | 99 | 8,290 | 0-68,326 |
| 21 | 104 | 10,707 | 0-226,331 | 99 | 4,396 | 0-43,960 |
| 28 | 105 | 4,522 | 0-111,030 | 97 | 0 | 0-32,530 |

**Table S3. Mean, SD and range of haemoglobin 0-42 days by treatment arm**

|  |  |  | AS |  |  | QN |  |  |
| --- | --- | --- | --- | --- | --- | --- | --- | --- |
| Days | **N** | **Mean** | **SD** | **Range** | **N** | **Mean** | **SD** | **Range** |
| 0 | 109 | 10.3 | 1.6 | 6.0-16.5 | 102 | 10.2 | 1.6 | 5.3-14.1 |
| 1 | 108 | 9.2 | 1.6 | 5.7-14.2 | 102 | 9.1 | 1.6 | 5.0-12.6 |
| 2 | 108 | 9.0 | 1.6 | 5.8-14.5 | 102 | 8.7 | 1.6 | 4.7-12.5 |
| 3 | 107 | 8.9 | 1.6 | 5.3-13.7 | 101 | 8.6 | 1.6 | 5.1-12.4 |
| 7 | 108 | 9.3 | 1.4 | 6-13.5 | 102 | 9.3 | 1.3 | 5.6-12.0 |
| 14 | 107 | 10.5 | 1.1 | 4.7-12.5 | 100 | 10.6 | 0.9 | 7.7-13.2 |
| 21 | 106 | 11.3 | 0.9 | 8.7-13 | 100 | 11.6 | 0.9 | 9.4-13.8 |
| 28 | 106 | 11.6 | 0.7 | 9.7-13.3 | 96 | 11.6 | 0.8 | 9.1-13.5 |
| 35 | 106 | 11.9 | 1.0 | 7.1-14.1 | 99 | 11.9 | 1.1 | 9.1-13.8 |
| 42 | 106 | 11.9 | 0.97 | 7.9-13.6 | 99 | 11.9 | 0.9 | 9.3-14.0 |

**Table S4. Linear regression using haemoglobin (g/dL) as outcome and patient as random effect for days 0-7**

|  | Coefficient | 95% CI | p-value |
| --- | --- | --- | --- |
| G6PD homo/hemizygous | -0.687 | -1.229, -0.145 | 0.013 |
| Sickle Cells Trait | 0.140 | -0.314, 0.594 | 0.545 |
| Male | 0.045 | -0.277, 0.368 | 0.784 |
| Age (years) | 0.1108 | 0.055, 0.161 | <0.001 |
| Malnourished | -0.379 | -0.734, -0.024 | 0.036 |
| [log] parasitaemia | -0.258 | -0.821, 0.305 | 0.369 |
| AS treatment | 0.3123 | -0.0021, 0.627 | 0.051 |
| LDH | -0.001 | -0.002, -0.001 | <0.001 |
| Palpable spleen | -0.431 | -0.749, -0.113 | 0.008 |
| [log] RESA | -0.353 | -0.445, -0.261 | <0.001 |
| [log] reticulocytes | -0.080 | -0.162, 0.002 | 0.055 |

**Table S5. Linear regression using haemoglobin (g/dL) as outcome and patient as random effect for days 7-28**

|  | Coefficient | 95% CI | p-value |
| --- | --- | --- | --- |
| G6PD homo/hemizygous | -0.081 | -0.455, 0.293 | 0.672 |
| Sickle Cells Trait | -0.028 | -0.346, 0.291 | 0.865 |
| Male | -0.175 | -0.397, 0.048 | 0.124 |
| Age (years) | -0.013 | -0.049, 0.023 | 0.472 |
| Malnourished | -0.171 | -0.417, 0.075 | 0.173 |
| [log] parasitemia | -0.273 | -0.663, 0.117 | 0.170 |
| AS treatment | 0.190 | -0.028, 0.408 | 0.087 |
| LDH | -0.003 | -0.003, -0.002 | < 0.001 |
| Palpable spleen | -0.219 | -0.439, 0.001 | 0.051 |
| [log] RESA | -0.201 | -0.247, -0.156 | < 0.001 |
| [log] reticulocytes | -0.452 | -0.531, -0.373 | < 0.001 |
