# Supplementary figures and images for: Post-treatment haemolysis in African children with hyperparasitaemic falciparum malaria; a randomized comparison of artesunate and quinine
Source: BMC Infect Dis. 2017 Aug 17;17:575. doi: 10.1186/s12879-017-2678-0 (PMC5561573; doi:10.1186/s12879-017-2678-0)

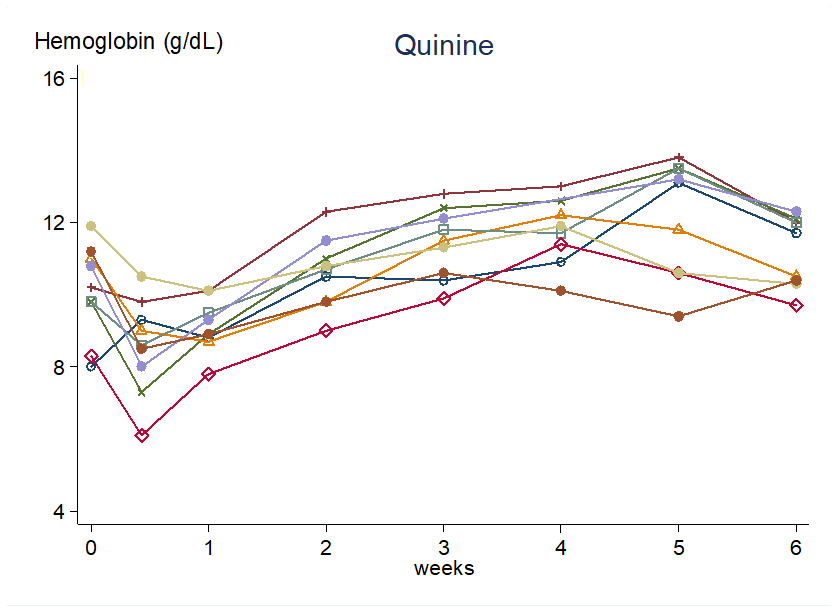

Supplement: Supplementary file 2 — Individual haemoglobin profiles of patients in the QN group with ≥10% reduction after day 7, n = 9 (TIFF 24 kb) [file 12879_2017_2678_MOESM2_ESM.tif]

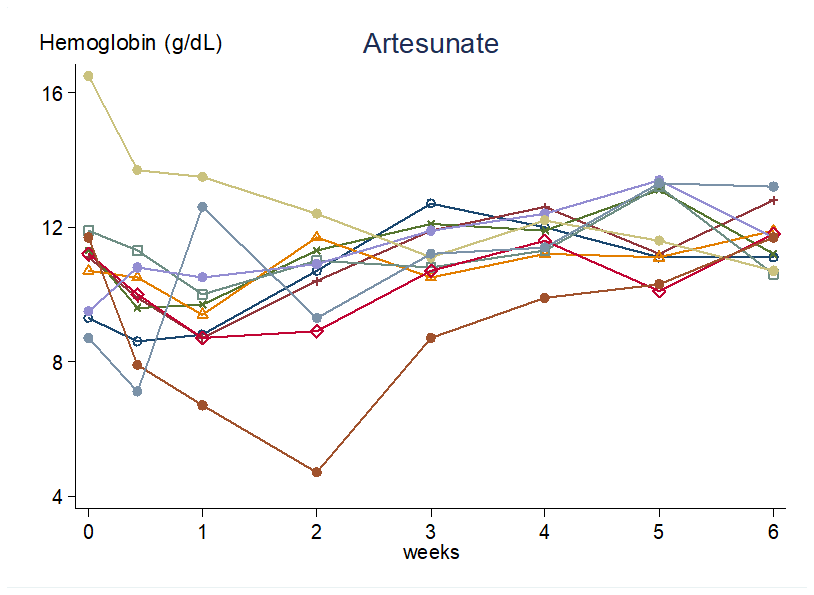

Supplement: Supplementary file 3 — Individual haemoglobin profiles of patients in the AS group with ≥10% reduction after day 7, n =10 (TIFF 64 kb) [file 12879_2017_2678_MOESM3_ESM.tif]
